# Inequalities in mortality among refugees and immigrants compared to native Danes – a historical prospective cohort study

**DOI:** 10.1186/1471-2458-12-757

**Published:** 2012-09-10

**Authors:** Marie Norredam, Maja Olsbjerg, Jorgen H Petersen, Knud Juel, Allan Krasnik

**Affiliations:** 1 Danish Research Centre for Migration, Ethnicity and Health, Department of Public Health, University of Copenhagen, Denmark, Øster Farimagsgade 5,Building 10, DK-1014, Copenhagen K, Denmark; 2 Department of Biostatistics, Department of Public Health, Faculty of Health Sciences, University of Copenhagen, Copenhagen, Denmark; 3 The National Institute of Public Health, University of Southern Denmark, Copenhagen, Denmark

**Keywords:** Immigrants, Refugees, Migration, Ethnicity, Inequity, Mortality

## Abstract

**Background:**

Comparisons of mortality patterns between different migrant groups, and between migrants and natives, are relevant to understanding, and ultimately reducing, inequalities in health. To date, European studies on migrants’ mortality patterns are scarce and are based solely on country of birth, rather than migrant status. However, mortality patterns may be affected by implications in relation to migrant status, such as health hazards related to life circumstances before and during migration, and factors related to ethnic origin. Consequently, we investigated differences in both all-cause and cause-specific mortality from cancer and cardiovascular disease among refugees and immigrants, compared with the mortality among native Danes.

**Methods:**

A register-based, historical prospective cohort design. All refugees (n = 29,139) and family-reunited immigrants (n = 27,134) who, between 1 January1993 and 31 December1999, were granted right of residence in Denmark were included and matched 1:4 on age and sex with native Danes. To identify deaths, civil registration numbers were cross-linked to the Register of Causes of Death (01.01.1994–31.12.2007) and the Danish Civil Registration System (01.01.1994–31.12.2008). Mortality rate ratios were estimated separately for men and women by migrant status and region of birth, adjusting for age and income and using a Cox regression model, after a median follow-up of 10–13 years after arrival.

**Results:**

Compared with native Danes, all-cause mortality was significantly lower among female (RR = 0.78; 95%CI: 0.71;0.85) and male (RR = 0.64; 95%CI: 0.59-0.69;) refugees. The rates were also significantly lower for immigrants: women (RR = 0.44; 95%CI: 0.38;0.51) and men (RR = 0.43; 95%CI: 0.37;0.51). Both migrant groups also had lower cause-specific mortality from cancer and cardiovascular diseases. For both all-cause and cause-specific mortality, immigrants generally had lower mortality than refugees, and differences were observed according to ethnic origin.

**Conclusions:**

Mortality patterns were overall advantageous for refugees and immigrants compared with native Danes. Research should concentrate on disentangling the reasons behind migrants’ health advantages, in order to enlighten future preventive public-health efforts, for the benefit of the entire population.

## Background

European studies [1-3] have shown lower all-cause mortality among migrants than among the native-born population, although specific risks vary between different ethnic groups. For cause-specific mortality, the risks differ between migrants and natives for specific disease categories. For example, cancer mortality is generally lower among migrants than among natives [4-6], whereas mortality from cardiovascular diseases (CVD) has shown a more contrasting picture [7-10]. Comparisons of mortality patterns between different migrant groups, and between migrants and natives, are relevant to understanding, and ultimately reducing, inequalities in health.

Immigrants and descendants make up 9.5% of the Danish population [11]. The focus of this study is on refugees and family-reunited immigrants. Since 1993, Denmark has received approximately 75,000 refugees and 105,000 family-reunited immigrants [12,13]. Refugees enter Denmark as quota refugees or spontaneous asylum seekers. Denmark receives approximately 500 quota refugees each year, under an agreement between the Danish State and the United Nations High Commissioner for Refugees (UNHCR), while asylum seekers arrive by their own means. Family-reunited immigrants arrive in Denmark independently and rely entirely on their families when establishing their new lives. In this paper, family-reunited immigrants are referred to as ‘immigrants’, while ‘migrants’ refers to all groups of foreign-born individuals.

To date, European studies on migrants’ mortality patterns are scarce. Those that exist use country of birth, self-assessed ethnicity or citizenship as a determinant, rather than migrant status (refugee vs. immigrant). However, mortality patterns may be affected by implications due to migrant status, such as health hazards related to life circumstances before and during migration, or factors related to ethnic origin. Consequently, we studied the effect of both migrant status and ethnic origin on all-cause mortality and cause-specific mortality from cancer and CVD, based on the unique Danish opportunities to use register-based national data.

## Methods

### Study cohort

The study cohort was obtained via the Danish Immigration Service’s statistics department. Migrants were included if they had obtained residence permits as refugees, or via family reunification in Denmark between 1.1.1993 and 31.12.1999. We identified 84,379 migrants. Individuals were excluded if they were below 18 years of age (n = 18,861) when they obtained their residence permits. Another 3,042 individuals were excluded due to missing civil registration numbers, or because their personal identification numbers appeared more than once in the sample. We also excluded 15 migrants due to irregularities in their registration of nationality. A Danish-born reference population was identified via Statistics Denmark. The comparison group was Danish-born residents with Danish-born parents, in order to avoid including descendants. Each of the family-reunited immigrant or refugee was age and sex matched with four Danish-born controls. Each control was selected randomly from all eligible controls without replacement, so each control was a control only once for a family-reunited immigrant or refugee. We matched 4:1 on an individual level on age and sex using a random sampling procedure. We were able to make a matching for all refugees resulting in 145 870 individuals: 29 174 refugees and 116 696 controls. Four of the family-reunited immigrants were missing a total of five controls due to difficulties with age matching because of outlying ages; accordingly, there were 33 287 family-reunited immigrants and 133 143 controls. The study cohort and matching procedure has previously been described in more detail [14].Baseline characteristics of the cohort on 31.12.1999 when inclusion was ended showed more males (55.6%) among refugees and conversely more females (64%) among immigrants’. Refugees and their Danish-born comparisons controls were 32.9 years old and immigrants and their Danish-born comparisons 27.5 years (see Table 1 stratified by sex). Refugees and their controls had been followed for a mean of about 8 years and immigrants and their comparisons for a mean of about 6 year. In this study we further excluded 6,188 migrants born in western countries and their corresponding Danish-born comparisons. Western countries does not include refugee producing areas like Former Yugoslavia, which was separated out due to their large numbers. The final study cohort comprised 29,139 refugees (native Danes:116,556) and 27,134 immigrants (native Danes:108,534). Migrants were consecutively censored on their first registered emigration date and were not included again if they later returned to Denmark. The follow-up time was thus defined for refugees and immigrants and their corresponding native Danes as the time from the commencement of the right of residence until the time of the first of the following events: a) death; b) end of study (31.12.2007/31.12.2008); or c) first emigration. As described below, the end of study for the analyses of all-cause mortality was based on data until 31.12.2008; and for cause-specific mortality until 31.12.2007. Updated characteristics of both study populations are shown in Table 1 (region of origin is shown for the population on 31.12.2007 only, due to the very small differences compared with the population on 31.12.2008).

**Table 1:** Characteristics of the study cohort distributed by sex and migrant status on respectively 31.12.2007 (cause-specific mortality analysis) and 31.12.2008 (all-cause mortality analysis)

**Study cohort on 31.12.2007**	**Female refugees**	**Danes**	**Male refugees**	**Danes**	**Female immigrants**	**Danes**	**Male immigrants**	**Danes**
**Regional of origin**	**% (n)**	**% (n)**	**% (n)**	**% (n)**	**% (n)**	**% (n)**	**% (n)**	**% (n)**
Asia	1.8 (238)		3.1 (509)		27.3 (4,996)		10.8 (955)	
East Europe	2.0 (258)		1.8 (297)		16.8 (3,100)		5.4 (481)	
Former Yugoslavia	57.2 (7,404)		49.2 (7,965)		5.7 (1,038)		9.8 (856)	
Iraq	11.3 (1,464)		19.5 (3,154)		6.5 (1,185)		2.0 (180)	
Middle East	10.3 (1,329)		10.0 (1,625)		27.8 (5,088)		47.3 (4,140)	
North Africa	15.7 (2,031)		14.5 (2,335)		9.8 (1,826)		15.0 (1,322)	
Sub-Saharan Africa	1.7 (225)		1.9 (305)		6.1 (1,121)		9.7 (846)	
Total	100 (12,949)	100 (51,796)	100 (16,190)	100 (64,760)	100 (18,354)	100 (73,416)	100 (8,780)	100 (35,118)
**Sex**	44.4 (12,949)	44.4 (51,796)	55.6 (16,190)	55.6 (64,760)	67.6 (18,354)	67.6 (73,416)	32.4 (8,780)	32.4 (35,118)
**Median age **at cohort closure 21.12.1999	**% (n)**	**% (n)**	**% (n)**	**% (n)**	**% (n)**	**% (n)**	**% (n)**	**% (n)**
Total deaths	3.7 (481)	4.7 (2,451)	4.0 (657)	5.2 (3,377)	0.9 (171)	1.9 (1,411)	1.6 (143)	3.3 (1,164)
Emigrations	13.6 (1,765)	3.8 (1,977)	14.3 (2,324)	5.0 (3,212)	17.6 (3,222)	6.0 (4,458)	22.9 (2,017)	6.6 (2,333)
Population at closure 31.12.2007	83.0 (10,703)	91.5 (47,368)	81.7 (13,209)	88.8 (58,171)	81.5 (14,961)	92.1 (67,547)	75.4 (6,620)	90.1 (31,621)
Events during follow-up till 31.12.2007	33.1 (26.5;42.9)	33.1 (26.5;42.9)	32.6 (26.4;41.0)	32.6 (26.4;41.0)	26.6 (22.2;33.1)	26.6 (22.2;33.1)	27.0 (23.4;32.5)	27.0 (23.4;32.4)
**Median age** at study end 31.12.2007	44.3 (37.1;54.2)	44.7 (37.7;54.5)	43.5 (37.0;52.2)	43.9 (37.6;52.4)	36.8 (32.1;43.4)	37.5 (32.8;44.1)	36.9 (32.8;42.2)	37.6 (33.8;43.3)
**Median follow-up in years**	12.1 (10.3;12.4)	12.1 (10.7;12.5)	11.9 (9.7;12.4)	12.1 (10.5;12.5)	10.6 (9.0;12.4)	10.9 (9.2;12.6)	10.5 (9.0;12.2)	10.8 (9.2;12.4)
**Events during follow-up till 31.12.2008**	**% (n)**	**% (n)**	**% (n)**	**% (n)**	**% (n)**	**% (n)**	**% (n)**	**% (n)**
Total deaths	4.1 (523)	5.3 (2,738)	4.5 (724)	5.8 (3,744)	1.1 (194)	2.2 (1,583)	1.7 (155)	3.7 (1,273)
Emigrations	14.2 (1,844)	4.0 (2,056)	15.2 (2,456)	5.2 (3,374)	18.2 (3,341)	6.2 (4,617)	23.8 (2,087)	6.9 (2,438)
Population at study end 31.12.2008	81.7 (10,582)	90.7 (47,002)	80.3 (13,010)	89.0 (57,642)	80.7 (14,819)	91.6 (67,216)	74.5 (6,538)	89.4 (31,407)
**Median age** at study end 31.12.2008	45.2 (37.9;55.1)	45.7 (38.7;55.5)	44.4 (37.8;53.1)	44.9 (38.5;53.4)	37.6 (32.9;44.3)	38.5 (33.7;45.0)	37.7 (33.6;43.0)	38.7 (34.7;44.3)
**Median follow-up in years**	13.1 (11.2;13.4)	13.1 (10.4;13.4)	12.9 (10.6;13.4)	13.1 (11.4;13.5)	11.6 (9.9;13.3)	11.9 (10.2;13.5)	11.4 (9.9;13.2)	11.7 (10.2;13.4)

### Data collection

The study cohort’s civil registration numbers were cross-linked to the Danish Register on Causes of Death, which contains data from the death certificates reported to the Danish National Board of Health. The register was updated to 31.12.2007. Causes of death were coded according to the International Classification of Diseases, tenth revision (ICD-10). The register has used the ICD-10 coding system since 01.01.1994, which represented a change from the ICD-8 coding system previously used. As the validity of the translations in the register from ICD-8 to ICD-10 is questionable, we only used ICD-10 diagnoses starting from 01.01.1994. All deaths in the cohort, which had cancer (C00-D09) and CVDs (I00-I25;I27,I30-I52) as the underlying death cause were identified. To increase follow-up time and thereby enlarge the sample size in the study of all-cause mortality, we cross-linked our cohort to population data from the Danish Civil Registration System, which contained data on all registered deaths (without causes of death) until 31.13.2008. In total, 10,934 in the cohort had died from 1.1.1994 to 31.12.2008, and 9,855 had died from 1.1.1994 to 31.12.2007, of whom 168 individuals lacked a registered cause of death.

We adjusted for region of origin according to the largest migrant groups in Denmark. We based our definition of geographical areas on WHO guidelines [15]: Asia, eastern Europe, former Yugoslavia, Iraq, Middle East, North Africa and Sub-Saharan Africa. We also adjusted for data on income using annual personal income based on wages from earnings and social transfers. Statistics Denmark obtains all income information from tax authorities. Information on income is updated annually on 31 December. Personal income was divided into three categories: Low (<€13,500/year), Middle (€13,500–40,300/year) and High (>€40,300/year). In total, 848 individuals did not have a registered income by 31.12.2007, including 113 refugees (native Danes: 224) and 368 immigrants (native Danes: 143); and 846 individuals did not have a registered income by 31.12.2008, including 113 refugees (native Danes: 224) and 368 immigrants (native Danes: 141). These individuals were all excluded from the analysis, which was therefore based on 29,026 refugees (native Danes: 116,322) and 26,766 immigrants (native Danes:108,391) for *cause-specific* mortality (31.12.2007); and on 29,026 refugees (native Danes: 116,322) and 26,766 immigrants (native Danes: 108,389) for *all-cause* mortality (31.12.2008).

According to the Danish Act on Processing of Personal Data all data subjects must be guaranteed confidentiality and anonymity. Therefore, individual level data are not openly available. Instead datasets and linkages between datasets are constructed at Statistics Denmark. Researchers may then access data using remote online access.

### Statistical analysis

We estimated the rates (RR) and 95% confidence intervals for refugees and immigrants compared with native Danes using a Cox regression model (in SAS version 9.1), which was adjusted separately for men and women. The Cox regression analysis implies continuous adjustment for age in the model. The analyses were performed similarly for both all-cause and cause-specific mortality. native Danes formed the reference group. The relative risk was analysed by migrant status and region of birth, and adjusted for income. The analyses take account of the time during which an individual has been in a certain income category, and allows for people to change from one income category to another over time. We assumed that the effect of income on mortality was the same for refugees, immigrants and native Danes. Before making this assumption, we analysed all interactions between migrant status, region of birth and income, and found no significant interactions. We studied unadjusted RR and then RR adjusted for income. As these separate analyses gave the same conclusion, Table 2 shows only the adjusted estimates. The Cox regression model allows comparison of the relative risk for refugees and immigrants compared with that for native Danes.

**Table 2:** Hazard ratios (RR) of sex specific all-cause mortality estimated by migrant status and region of birth, adjusting for age and income. Danish born controls form the reference group

	**Female refugees**	**n = 12,890**			**Male refugees**	**n = 16,136**		
**Region of origin**	**RR**	**95%CI**	**n**	**p-value**	**RR**	**95%CI**	**n**	**p-value**
Asia	0.90	(0.50 – 1.63)	11	0.72	0.41	(0.24 – 0.71)	13	0.002
Eastern Europe	0.75	(0.36 – 1.58)	7	0.45	0.59	(0.31 – 1.14)	9	0.11
Former Yugoslavia	0.81	(0.73 – 0.90)	375	<0.0001	0.70	(0.63 – 0.77)	502	<0.0001
Iraq	0.62	(0.44 – 0.87)	33	0.005	0.45	(0.35 – 0.58)	66	<0.0001
Middle East	0.53	(0.38 – 0.74)	35	0.0002	0.53	(0.41 – 0.68)	64	<0.0001
North Africa	0.84	(0.61 – 1.16)	38	0.29	0.76	(0.58 – 1.00)	52	0.052
Sub-Saharan Africa	2.70	(1.28 – 5.66)	7	0.008	0.52	(0.20 – 1.39)	4	0.19
**All**	**0.78**	**(0.71 – 0.85)**	**506**	**<0.0001**	**0.64**	**(0.59 – 0.69)**	**710**	**<0.0001**
	**Female immigrants**	**n = 18,132**			**Male immigrants**	**n = 8,634**		
**Region of origin**	**RR**	**95%CI**	**n**	**p-value**	**RR**	**95%CI**	**n**	**p-value**
Asia	0.35	(0.26 – 0.48)	39	<0.0001	0.40	(0.28 – 0.57)	29	<0.0001
Eastern Europe	0.47	(0.35 – 0.64)	42	<0.0001	0.62	(0.37 – 1.03)	15	0.064
Former Yugoslavia	0.50	(0.32 – 0.79)	19	0.002	0.58	(0.39 – 0.88)	24	0.009
Iraq	0.27	(0.12 – 0.61)	6	<0.001	0.45	(0.20 – 0.99)	6	0.048
Middle East	0.45	(0.34 – 0.59)	51	<0.001	0.36	(0.27 – 0.49)	46	<0.0001
North Africa	0.31	(0.16 – 0.60)	9	<0.001	0.30	(0.17 – 0.52)	13	<0.0001
Sub-Saharan Africa	1.12	(0.69 – 1.84)	16	0.64	0.83	(0.49 – 1.41)	14	0.49
**All**	**0.44**	**(0.38 – 0.51)**	**182**	**<0.0001**	**0.43**	**(0.37 – 0.51)**	**147**	**<0.0001**

Analyses are based on deaths among the population (n) with a registered income from 1.1.1994 – 31.12.2008.

## Results

Table 1 shows median age at study end, median follow-up time and events during follow-up for the cohort on 31.12.2007 (for the cause-specific mortality analysis) and on 31.12.2008 (for the all-cause mortality analysis), respectively. Ethnic sub-groups are shown only for the 2007 population. Approximately half the refugees in the cohort came from the former Yugoslavia, while immigrants originated mainly from the Middle East and Asia.

Table 2 shows the rates of sex-specific all-cause mortality estimated by migrant status and region of birth, and adjusted for age and income. Overall, both refugee women (RR = 0.78; 95%CI: 0.71; 0.85) and men (RR = 0.64; 95%CI: 0.59;0.69) had significantly lower all-cause mortality than native Danes. Differences according to ethnicity were observed: refugee women from the former Yugoslavia, Iraq and the Middle East had a significantly lower rates, and those from Sub-Saharan Africa had a significantly higher rate, compared with native Danes. All other regions of origin showed no significant differences compared with native Danes. Among refugee men, individuals from Asia, the former Yugoslavia, Iraq and the Middle East had significantly lower all-cause mortality compared with native Danes, while all other sub-groups showed no significant differences compared with native Danes. Overall, immigrant women (RR = 0.44; 95%CI: 0.38;0.51) and men (RR = 0.43; 95%CI: 0.37; 0.51) also showed significantly decreased all-cause mortality rates compared with native Danes. All ethnic sub-groups also showed significantly lower rates, apart from women from Sub-Saharan Africa, and men from eastern Europe and Sub-Saharan Africa, whose rate did not differ significantly.

Table 3 shows the rates of sex-specific cancer mortality estimated by migrant status and region of birth, and adjusted for age and income. Overall, refugee women (RR = 0.75; 95%CI: 0.63; 0.88) showed significantly lower mortality from cancer compared with native Danes, whereas the result for refugee men (RR = 0.86; 95%CI: 0.73; 1.00) was only borderline significant. Analyses according to region of origin showed significantly lower rates for refugee women from Iraq, the Middle East and North Africa. All other sub-groups showed no significant differences compared with native Danes, including refugee women from the former Yugoslavia, who accounted for the majority of deaths, but showed only a non-significant tendency for lower cancer mortality. For refugee men, significantly lower cancer mortality was found for individuals from Iraq. Apart from this, all other regions of origin showed no significant differences in rate compared with native Danes, including refugee men from the former Yugoslavia, who accounted for the majority of deaths. Overall, immigrant women (RR = 0.36; 95%CI: 0.26;0.48) and men (RR = 0.5; 95%CI: 0.39; 0.78) also had significantly reduced mortality from cancer compared with native Danes. Analyses according to region of origin showed significantly lower rates for both immigrant women and men from Asia and the Middle East, compared with native Danes. All other sub-groups showed non-significant tendencies towards lower cancer mortality.

**Table 3:** Hazard ratios (HR) of sex specific cancer mortality estimated by migrant status and region of birth, adjusting for age and income

	**Female refugees**	**n = 12,890**			**Male refugees**	**n = 16,136**		
**Region of origin**	**RR**	**95%CI**	**n**	**p-value**	**RR**	**95%CI**	**n**	**p-value**
Asia	0.50	(0.12 – 1.99)	2	0.32	0.15	(0.02 – 1.08)	1	0.059
Eastern Europe	1.04	(0.33 – 3.22)	3	0.95	0.32	(0.05 – 2.29)	1	0.25
Former Yugoslavia	0.89	(0.75 – 1.07)	139	0.20	1.01	(0.85 – 1.20)	167	0.89
Iraq	0.33	(0.15 – 0.73)	6	0.006	0.35	(0.19 – 0.66)	10	0.001
Middle East	0.34	(0.16 – 0.71)	7	0.004	0.76	(0.49 – 1.19)	20	0.22
North Africa	0.41	(0.18 – 0.92)	6	0.029	0.64	(0.32 – 1.28)	8	0.20
Sub-Saharan Africa	-	-	0	-	1.57	(0.39 – 6.28)	2	0.52
**All**	**0.75**	**(0.63 – 0.88)**	**163**	**0.0005**	**0.86**	**(0.73 – 1.00)**	**209**	**0.051**
	**Female immigrants**	**n = 18,132**			**Male immigrants**	**n = 8,634**		
**Region of origin**	**RR**	**95%CI**	**n**	**p-value**	**RR**	**95%CI**	**n**	**p-value**
Asia	0.23	(0.12 – 0.47)	8	<0.0001	0.38	(0.17 – 0.85)	6	0.018
Eastern Europe	0.64	(0.39 – 1.05)	16	0.078	0.43	(0.11 – 1.73)	2	0.23
Former Yugoslavia	0.46	(0.19 – 1.11)	5	0.084	1.22	(0.63 – 2.35)	9	0.56
Iraq	0.17	(0.02 – 1.21)	1	0.076	0.99	(0.32 – 3.08)	3	0.99
Middle East	0.31	(0.17 – 0.59)	10	0.0003	0.42	(0.22 – 0.82)	9	0.01
North Africa	-	-	0	-	0.36	(0.12 – 1.12)	3	0.076
Sub-Saharan Africa	0.78	(0.25 – 2.41)	3	0.66	1.23	(0.40 – 3.84)	3	0.71
**All**	**0.36**	**(0.26 – 0.48)**	**43**	**<0.0001**	**0.55**	**(0.39 – 0.78)**	**35**	**0.0007**

Danish born controls form the reference group. Analyses are based on deaths among the population (n) with a registered income from 1.1.1994 – 31.12.2007.

Table 4 shows the rates of sex-specific cardiovascular disease (CVD) mortality, estimated by migrant status and region of birth, and adjusted for age and income. Overall, compared with native Danes, refugee women showed only borderline significant differences in mortality from CVD (RR = 0.80; 95%CI: 0.62;1.03), while refugee men had a significantly lower mortality from CVD (RR = 0.68; 95%CI: 0.55;0.84). Analyses according to region of origin showed no significant differences for refugee women from the various sub-groups, compared with native Danes. This was also the case for refugee men, apart from men from the former Yugoslavia and the Middle East, whose mortality was significantly lower. Overall, compared with native Danes, immigrant women (RR = 0.39; 95%CI: 0.25;0.61) and men (RR = 0.57; 95%CI: 0.38; 0.84) had significantly decreased mortality from CVD. Analyses according to region of origin showed lower mortality for all sub-groups compared with native Danes, albeit only significantly for immigrant women from Asia and eastern Europe, but otherwise there were no significant differences in rates. For immigrant men, no significant differences in rates on a sub-group level were found, compared with native Danes, apart from men from Asia, who had a significantly lower rate.

**Table 4:** Hazard ratios (RR) of sex specific cardiovascular (CVD) mortality estimated by migrant status and region of birth, adjusting for age and income

	**Female refugees**	**n = 12,890**			**Male refugees**	**n = 16,136**		
**Region of origin**	**RR**	**95%CI**	**n**	**p-value**	**RR**	**95%CI**	**n**	**p-value**
Asia	0.67	(0.09 – 4.75)	1	0.68	-	-	0	-
Eastern Europe	-	-	0	-	1.48	(0.47 – 4.60)	3	0.50
Former Yugoslavia	0.83	(0.62 – 1.11)	51	0.205	0.73	(0.58 – 0.93)	82	0.0102
Iraq	1.31	(0.65 – 2.63)	8	0.45	0.68	(0.40 – 1.19)	13	0.18
Middle East	0.78	(0.37 – 1.65)	7	0.51	0.43	(0.21 – 0.86)	8	0.017
North Africa	0.19	(0.03 – 1.32)	1	0.090	0.77	(0.35 – 1.73)	6	0.53
Sub-Saharan Africa	-	-	0	-	-	-	0	-
**All**	**0.80**	**(0.62 – 1.03)**	**68**	**0.0861**	**0.68**	**(0.55 – 0.84)**	**112**	**0.0003**
	**Female immigrants**	**n = 18,132**			**Male immigrants**	**n = 8,634**		
**Region of origin**	**RR**	**95%CI**	**n**	**p-value**	**RR**	**95%CI**	**n**	**p-value**
Asia	0.34	(0.13 – 0.91)	4	0.031	0.25	(0.08 – 0.77)	3	0.0159
Eastern Europe	0.31	(0.11 – 0.83)	4	0.02	0.72	(0.23 – 2.29)	3	0.58
Former Yugoslavia	0.69	(0.26 – 1.85)	4	0.45	0.52	(0.17 – 1.64)	3	0.26
Iraq	-	-	0	-	0.88	(0.22 – 3.54)	2	0.86
Middle East	0.53	(0.26 – 1.08)	8	0.08	0.49	(0.24 – 1.00)	8	0.049
North Africa	0.31	(0.04 – 2.20)	1	0.24	0.88	(0.37 – 2.15)	5	0.79
Sub-Saharan Africa	-	-	0	-	2.06	(0.67 – 6.51)	3	0.21
**All**	**0.39**	**(0.25 – 0.61)**	**21**	**< 0.0001**	**0.57**	**(0.38 – 0.84)**	**27**	**0.005**

Danish born controls form the reference group. Analyses are based on deaths among the population (n) with a registered income from 1.1.1994 – 31.12.2007.

## Discussion

### Key findings

Our results show that refugees and immigrants, divided by migrant status, had significantly lower all-cause and cause-specific mortality compared with native Danes, apart from the borderline significant result for cancer mortality for refugee men, and CVD mortality for refugee women. When stratified by ethnic group, results for both all-cause mortality and cause-specific mortality showed significantly reduced rates, or no differences, among migrants compared with native Danes, apart from Sub-Saharan refugee women, who had a significantly increased all-cause mortality rate. 

### Methodological strengths and limitations

The unique Danish national registers linking data on migration to national death statistics data that were used in our study make it a valuable contribution to the literature. We were able to identify a large cohort of refugees and immigrants based on specific information on migrant type from the immigration authorities. The design also allowed us to calculate all-cause and cause-specific mortality over 10–13 years’ follow-up, and enabled us to compare directly with a matched group of native Danes. Lastly, we could divide refugees and immigrants according to seven geographical sub-groups.

There are several factors that may have influenced our results. Firstly, although the study was based on a relatively large cohort of migrants, absolute numbers of deaths became somewhat small when specific diseases were investigated, and we stratified according to region of origin and migrant status. Nevertheless, despite this power problem, estimates from our analyses were still consistent and significant. However, further stratification according to specific cancer types and CVD was limited. Secondly, data from the Register on Causes of Death at the National Board of Health is lacking because during the study period the register did not receive death certificates for 3-4% of the deaths registered in the Population Register at Statistics Denmark. Thirdly, 1.7% (168/9,855) of all death certificates stated that the cause of death was ‘unknown’. Consequently, there may be differences between migrants and native Danes regarding the distribution of these ‘unknown’ causes of death, but these differences are not likely to be substantial, as the 168 deaths are rather equally distributed between migrants and native Danes. Fourthly, our results may be affected by registered or unregistered re-migration, which would skew the estimates. We did not know the extent of unregistered emigrations in our cohort, but only the number of registered first-time emigrations, which was higher among migrants than among native Danes (Table 1). Accordingly, we did not know the rates of ill and healthy individuals in this group. Consequently, we cannot exclude that bias due to unregistered or registered re-migration may have affected our results by either inflating or underestimating them. Deaths abroad of individuals with a Danish personal identification number are reported to the Danish authorities on an irregular basis; however, death certificates issued abroad and reported to the Register of Causes of Death were automatically excluded due to validity problems. We controlled for income, which is considered the most valid proxy for socioeconomic status (SES) for migrants in a Danish context. However, personal income may inadequately reflect SES especially among elderly and family reunified individuals. Education would have been a more stable indicator of SES, but education is not registered in a valid and consistent way for migrants in Denmark.

### All-cause mortality

Other studies have also documented reduced all-cause mortality rates among non-western migrants compared with natives, although variations according to ethnic origin occurred [1-3,16]. Our results are also supported by data from Statistics Denmark that reports an all-cause mortality that is reduced by 25% and 32% for men and women of non-western migrant origin, respectively. There are several possible explanations for this [17]. (i) The selection of healthy individuals into migration; ‘the healthy migrant effect. (ii) Beneficial health-related behaviours from countries of origin might persist and contribute to our results. For example, a German study documented that low all-cause mortality among migrant Turks extended to descendants [3]. (iii) The previously mentioned re-migration bias, implying that critically ill individuals return to their country of origin, may have also contributed to our results. The counter argument is that critically ill individuals will wish to stay close to well-functioning healthcare services, which the host country is more likely to offer. (iv) Differences in genetic susceptibility to diseases may account for our results. (v) Better access to quality healthcare in host countries would improve diagnosis and treatment and subsequently mortality. Stirbu et al. [16] documented differences in avoidable mortality, which suggests that although migrants may benefit from existing services, there is room for further quality improvement within specific areas such as infectious diseases, diabetes, hypertension and asthma. All these possible explanations are likely to interact, and the extent to which they explain our results is unknown.

Our results were less pronounced for refugees than for immigrants; and for individuals from Sub-Saharan Africa compared with those originating from other regions. These groups may have been more at risk of health problems before and during migration and, accordingly, ‘the healthy migrant’ effect would be less relevant in their case. A Danish study [18] found that among a group of newly arrived quota refugees, 65% had one or more severe somatic health problems, ranging from liver carcinoma to tuberculosis and diabetes. Refugees may therefore be in special need of screening programmes and targeted health services. In Denmark, medical screening is provided for asylum seekers, but not for quota refugees or refugees who are family-reunited.

### Cause-specific mortality

For *cancer mortality*, several other studies have shown reduced mortality from cancer among migrants compared with natives, although differences in cancer mortality vary according to cancer type [4-6,19]. Thus, cancers with high mortality in non-western countries, such as cancers of the liver, oesophagus and stomach, continue to exhibit high mortality among non-western migrants in European countries, whereas low-mortality cancers in non-western countries, including lifestyle-related cancers such as breast cancer, lung cancer and colorectal cancer, also exhibit low mortality among non-western migrants in European countries. Our findings of low cancer mortality among migrants thus seem to reflect differences in cancer incidence patterns. Furthermore, studies of the incidence of lung, colorectal, breast and gynaecological cancers in our cohort revealed low incidences among migrants [20]. Patterns of low overall cancer incidence are in accordance with Danish and international literature on exposure to cancer-related risk factors, which shows that alcohol consumption and exposure to tobacco are generally lower among first-generation migrants compared with natives [21-23]. Nevertheless, the protective effect of being a migrant in relation to lifestyle-related cancers appears to wear off over time with new generations, most likely due to new environmental exposures and changes in lifestyle [5].

For *CVD mortality* among migrants compared with native populations, the literature shows contrasting findings [2,8,9]. In support of our study, recent data from Statistics Denmark shows lower mortality from CVD among non-western migrants than among native Danes, both for men and women [24]. Our results are seemingly conflicting as studies show that the incidence of CVD is higher among non-western migrants in several European countries, including Denmark [25-28]. What may then explain our findings: (i) the CVD morbidity pattern is supported by studies on risk factors showing a higher prevalence of obesity, diabetes and hypertension in some migrant groups, although this is somewhat outbalanced by fewer smokers and lower alcohol consumption. However, we do not know whether migrants show more excessive risk behaviour than native citizens do after CVD diagnosis; (ii) genetic differences may play an as yet unexplored role; however, a recent European study documented variations in mortality from circulatory disease between similar migrant groups across six countries, indicating that the role of genetics was unlikely alone [7]; (iii) research generally documents more problems with access to diagnosis and treatment for migrants compared with native citizens, as demonstrated in a recent study suggesting that some migrants do not receive adequate medical treatment with beta-blockers after a first acute myocardial infarction (AMI) compared with native Danes [29]. The authors concluded that lower socioeconomic status, communication problems between doctor and patient, and doctors' attitudes towards migrants, may explain the differences.

## Conclusion

Overall, the conclusion of our study is encouraging in relation to migrants’ health. However, more knowledge is needed to disentangle and understand the biological and lifestyle-related mechanisms that unfold during transitions from low-income to high-income countries. Indeed, majority populations may also benefit from such research. Moreover, to prevent ethnic inequalities in mortality becoming more disadvantageous in the future, public health authorities should discourage the further adoption of unhealthy risk behaviour.

## Competing interests

The authors declare that they have no competing interests.

## Authors’contributions

MN: has made substantial contributions to conception and design, acquisition of data, analysis and interpretation of data and drafting the manuscript. MO: has made substantial contributions to design and statistical analysis and interpretation of data and revised the manuscript critically. JHP: has made substantial contributions to design and statistical analysis and interpretation of data and revised the manuscript critically. KJ: has made substantial contributions to conception and design and interpretation of data and revised the manuscript critically. AK: has made substantial contributions to conception and design and interpretation of data and revised the manuscript critically. All authors read and approved the final manuscript.

## Pre-publication history

The pre-publication history for this paper can be accessed here:

http://www.biomedcentral.com/1471-2458/12/757/prepub
